# Critical amino acids in the TM2 of EAAT2 are essential for membrane‐bound localization, substrate binding, transporter function and anion currents

**DOI:** 10.1111/jcmm.16212

**Published:** 2021-02-01

**Authors:** Dongmei Mai, Rongqing Chen, Ji Wang, Jiawei Zheng, Xiuping Zhang, Shaogang Qu

**Affiliations:** ^1^ Department of Neurology Nanfang Hospital Southern Medical University Guangzhou China; ^2^ Key Laboratory of Mental Health of the Ministry of Education Southern Medical University Guangzhou China; ^3^ Guangdong‐Hong Kong‐Macao Greater Bay Area Center for Brain Science and Brain‐Inspired Intelligence Guangzhou China; ^4^ Department of Neurobiology School of Basic Medical Sciences Southern Medical University Guangzhou China; ^5^ Teaching Center of Experimental Medicine School of Basic Medical Sciences Southern Medical University Guangzhou China

**Keywords:** alanine‐scanning mutation, excitatory amino acid transporter 2, glutamate, transmembrane domain 2, transporter activity

## Abstract

Excitatory amino acid transporter 2 (EAAT2), the gene of which is known as solute carrier family 1 member 2 (*SLC1A2*), is an important membrane‐bound transporter that mediates approximately 90% of the transport and clearance of l‐glutamate at synapses in the central nervous system (CNS). Transmembrane domain 2 (TM2) of EAAT2 is close to hairpin loop 2 (HP2) and far away from HP1 in the inward‐facing conformation. In the present study, 14 crucial amino acid residues of TM2 were identified via alanine‐scanning mutations. Further analysis in EAAT2‐transfected HeLa cells in vitro showed that alanine substitutions of these residues resulted in a decrease in the efficiency of trafficking/targeting to the plasma membrane and/or reduced functionality of membrane‐bound, which resulted in impaired transporter activity. After additional mutations, the transporter activities of some alanine‐substitution mutants recovered. Specifically, the P95A mutant decreased EAAT2‐associated anion currents. The Michaelis constant (K_m_) values of the mutant proteins L85A, L92A and L101A were increased significantly, whereas R87 and P95A were decreased significantly, indicating that the mutations L85A, L92A and L101A reduced the affinity of the transporter and the substrate, whereas R87A and P95A enhanced this affinity. The maximum velocity (Vmax) values of all 14 alanine mutant proteins were decreased significantly, indicating that all these mutations reduced the substrate transport rate. These results suggest that critical residues in TM2 affect not only the protein expression and membrane‐bound localization of EAAT2, but also its interactions with substrates. Additionally, our findings elucidate that the P95A mutant decreased EAAT2‐related anion currents. Our results indicate that the TM2 of EAAT2 plays a vital role in the transport process. The key residues in TM2 affect protein expression in the membrane, substrate transport and the anion currents of EAAT2.

## 1 INTRODUCTION

Glutamate is the most prevalent excitatory amino acid neurotransmitter in the mammalian central nervous system (CNS) and is necessary for virtually all normal brain functioning, including sensory processing, motor output, neural plasticity and long‐term potentiation.^1^ The extracellular concentration of glutamate is kept at a relatively low level to prevent excessive activation of glutamatergic receptors, which can lead to excitotoxicity and cellular death.^2^ Excitotoxicity caused by high concentrations of extracellular glutamate is related to many CNS diseases, such as Parkinson's disease,^3^ Alzheimer's disease,^4^ epilepsy,^5^ traumatic brain injury^6^ and amyotrophic lateral sclerosis.^7^ In mammals, the clearance of glutamate from synapses is dependent on glutamatergic transporters; in prokaryotes, transporters are responsible for the absorption of nutrients such as aspartic acid and glutamate.^8^ Therefore, transporters are fundamentally critical to cellular function across species.

Eukaryotic glutamatergic transporters are mainly composed of high‐affinity glutamatergic transporters (eg excitatory amino acid transporters, EAATs) and low‐affinity glutamatergic transporters (eg vesicular glutamate transporters, vGLUTs). Five EAAT subtypes have been cloned and consist of EAAT1 (also known as glutamate aspartate transporter [GLAST]),^9^ EAAT2 (also known as glial glutamate transporter 1 [GLT‐1]),^10^ EAAT3 (also known excitatory amino acid carrier 1 [EAAC1]),^11^ EAAT4^12^ and EAAT5.^13^ There is a 50% homology in the amino acid sequences among these five subtypes,^5^, ^14^, ^15^ among which the glutamatergic transporter, EAAT2/GLT‐1, is mainly expressed in astrocytes.^16^, ^17^ In addition, EAATs are electrogenic and the EAAT transport cycle comprises co‐transport of three sodium ions with glutamate, accompanied by a reverse transport of one potassium ion.^18^, ^19^ vGLUTs, which are mainly located on synaptic vesicles, specifically bind glutamate, absorb it into vesicles and promote its extracellular release into synapses, which is closely related to the generation of normal excitatory synaptic conduction.^20^ Therefore, the production, maintenance and termination of normal excitatory synaptic conduction require the synergistic effects of EAATs and vGLUTs. Dysregulation of GLT‐1 expression and function may play an important role in excitotoxicity and related neuropathogenesis, as approximately 90% of glutamatergic transport is mediated by EAAT2.^2^, ^21^ A previous study has reported that EAAT2 dysfunction or decreased expression has been found in acute and chronic neurodegenerative diseases.^22^ In addition, evidence in animal models of disease suggests that up‐regulation of EAAT2 is a potential therapeutic strategy to prevent excitotoxicity.^23^, ^24^, ^25^

In 2004, Yernool et al. proposed the first crystal structure of a glutamatergic transporter homologue from *P horikoshii* (Glt_Ph_).^26^ The sodium‐coupled aspartic acid transporter, Glt_Ph_, is homologous to eukaryotic glutamic acid transporter and exhibits both inward‐facing and outward‐facing conformations (ie cytoplasm‐facing and extracellular‐facing conformations, respectively), in addition to intermediate conformations.^26^, ^27^, ^28^ Glt_Ph_ is a trimer in which each of its monomers represents an independent functional unit.^26^ In 2017, the crystal structures of the outward conformation and intermediate conformation of EAAT1 were characterized, which have provided an excellent model to further elucidate the structure and function of eukaryotic glutamatergic transporters.^29^ However, the crystal structure of the inward conformation of EAAT1 has not been characterized. Each monomer of glutamatergic transporters contains eight transmembrane domains (TMs 1‐8) and two opposite hairpin loops (HP1 and HP2), among which HP1 is located between TM6 and TM7, and HP2 is located between TM7 and TM8. It has been demonstrated that TMs 1‐6 form the shell of each transporter monomer, whereas HP1, TM7, HP2 and TM8 are involved in the formation of the substrate‐binding pocket.^30^, ^31^ These subunits are connected to form a scaffold surrounding a highly conserved dense core region.^32^ After substrate binding, the transport core—composed of HP1, TM7, HP2 and TM8—moves inward (intracellularly) relative to the other part of the transporter, forming a cytoplasmic conformation.^19^ Thus in the inward‐facing conformation, the TM2 of a glutamatergic transporter is far from HP1,^33^ but is in close apposition to HP2.^19^ HP1 and HP2 function as the inner and outer gates, respectively, of glutamatergic transporters.^19^, ^26^, ^27^, ^31^ HP2 openings expose substrate‐binding sites^27^, ^31^ and—following substrate binding to co‐transported ions—close and concomitantly block these binding sites. After HP2 is closed, the transport core moves to the cytoplasm with a piston‐like motion, which enables the cytoplasm‐facing HP1 to open.^27^ Potassium ions then facilitate the glutamatergic transporter to be redirected from an inward‐ to outward‐facing conformation, thus completing a transport cycle. Although the TM2 of each glutamatergic transporter is close to the substrate‐binding zone and may participate in or co‐ordinate the transport of substrates, the specific mechanisms of TM2 in this process remain unclear.

Transmembrane domain 2s are highly conserved between Glt_Ph_ and EAATs in their roles in maintaining balance of their corresponding transporter cores during translocation.^26^ Moreover, TM2 mutations in EAAT1 change the permeability properties of anions without affecting glutamate transfer. In addition, it has been demonstrated that the extracellular edge of TM2 is in close apposition to membrane‐related domains that affect glutamatergic transport.^34^ The ionic osmotic pathways of various ion channels are composed of amino acid residues and have polar side chains such as serine, threonine and tyrosine.^35^, ^36^ In EAAT1, both of the ends of TM2 are positively charged and contain a large number of polar residues that are highly conserved between transporter subtypes. It has been demonstrated that EAATs act as both transporters and anion‐selective ion channels at synapses.^37^ The anionic channels of EAATs regulate neuronal excitability, and functionally acquired mutations of these proteins cause ataxia and epilepsy.^38^, ^39^, ^40^, ^41^ EAAT‐related openings and closings of anionic channels depend on the combination of sodium and glutamic acid. Therefore, structural elements must exist to enable coupling between substrate‐binding sites and anion‐channel openings; however, the structural components for this coupling have not yet been identified.^42^ According to the positions and properties of residues in TM2, we hypothesized that the structure of this domain may represent part of the EAAT2 anionic permeation pathway.

Genetic studies have shown that mutations (Gly82Arg, Leu85Pro) in the *slc1a2* gene is involved in the pathogenesis of epilepsy, and these two residues are located in TM2 regions of EAAT2.^43^, ^44^ In addition, it has been shown that the molecular determinants of anionic permeation via TM2 may be closely located to the carboxyl‐terminal region of EAAT2 and may be related to substrate binding and transport.^34^ Therefore, in the present study, we investigated the structure‐function relationship of TM2 in EAAT2 in HeLa cells in vitro. We employed alanine‐scanning mutations of TM2 residues within EAAT2, which revealed 14 critical amino acid residues related to EAAT2 uptake activity. Combined with cell‐surface biotinylation assays and kinetic analyses, we found that these critical residues of TM2 affected the plasma‐membrane localization and glutamatergic uptake of EAAT2 in HeLa cells in vitro. Moreover, through electrophysiological experiments, we found that the P95A mutant decreased EAAT2‐associated anion currents.

## 2 MATERIALS AND METHODS

### 2.1 Site‐directed mutagenesis

The KOD‐Plus‐Mutation Kit (TOYOBO, Osaka, Japan) was used to generate all mutants, as described previously.^45^, ^46^ The pBluescript SK (−) vector (Stratagene, La Jolla, CA, USA) containing SLC1A2 cDNA (from *Rattus norvegicus*) was used as the template for mutagenesis using antisense primers of mutants. Mutant DNA was then transformed into *Escherichia coli* TOP10 cells. Finally, all mutation sequences were verified by full‐length sequencing (Sangon Biotech).

### 2.2 Cell culture and plasmid transfection of HeLa cells

Cell culture and plasmid transfection of HeLa Cells were performed as described previously, with slight modifications.^47^ HeLa cells were cultured and grown at 37°C and 5% CO_2_ in DMEM basic medium (Gibco) supplemented with 8% foetal bovine serum (ExCell Biology), 200 U/mL penicillin and 200 μg/mL streptomycin (Beyotime Biotechnology). HeLa cells were seeded in 12‐well (2.0 × 10^5^ cells/well) or 24‐well (5 × 10^4^ cells/well) plates and were transfected with recombinant vaccinia virus for 30 minutes, followed by WT EAAT2 or mutant plasmid transfection using Lipo6000™ transfection reagent according to the manufacturer's instructions. Cells were incubated for 16‐24 hours at 37°C prior to transporter assays or cell‐surface biotinylation assays.

### 2.3 D‐[^3^H]‐aspartate uptake in HeLa cells

HeLa cells were grown in 24‐well plates, and D‐[^3^H]‐aspartate uptake was performed after transfection for 16‐18 hours. Uptake of D‐[^3^H]‐aspartate was performed as described previously.^48^, ^49^ The cells were washed once with choline chloride (ChCl) solution (4 mol/L ChCl, 1 mol/L MgSO_4_, 2 mol/L CaCl_2_, 1 mol/L Kpi [1 mol/L K_2_HPO_4_, 1 mol/L KH_2_PO_4_] [pH 7.4]) and were then incubated with 0.4 μCi (0.15 μ mol/L) of D‐[^3^H]‐aspartate (PerkinElmer) diluted with NaCl solution (4 mol/L NaCl, 1 mol/L MgSO_4_, 2 mol/L CaCl_2_, 1 mol/L KPi [pH 7.4]) at room temperature for 10 minutes. Cold NaCl solution was added twice to terminate the reaction. Cells were dissolved with 1% SDS and transferred to the sample tubes. Then, 3 mL of scintillant solution was added to each tube and was thoroughly mixed, and the counts per minute (CPM) value of ^3^H was detected by a liquid scintillation counter (PerkinElmer).

### 2.4 Kinetic assays

HeLa cells were seeded in 24‐well plates and incubated with 154 nmol/L of D‐[^3^H]‐aspartate at room temperature for 10 minutes, which was diluted by final unlabelled D‐aspartic acid at concentrations of 1, 3, 10, 50, 100, 500, 1000 and 1500 μmol/L. The K_m_ and V_max_ values were determined by fitting the data to the Hill equation using Origin 7.5 software (Northampton, MA, USA). V_max_ was expressed as a percentage of that of WT EAAT2. Each experiment was repeated three times.

### 2.5 Cell‐surface biotinylation assays

Cell‐surface expression levels of WT EAAT2 and EAAT2 mutants were detected by EZ‐Link Sulfo‐NHS‐SS‐biotin (Thermo Fisher Scientific). WT EAAT2 or EAAT2 mutants were transfected into HeLa cells in 12‐well plates using Lipo6000 (Beyotime Biotechnology). All steps were performed on ice. After 16‐18 hours of transfection, cells were washed twice with 1 mL of cold PBS (pH 8.0) per well. Then, 500 μL of NHS‐SS‐biotin (0.5 mg/mL in PBS, pH 8.0) was incubated twice (20 minutes each time). After biotinylation, cells were washed twice with 100 mmol/L glycine for 20 minutes and unreacted biotin reagent was removed. Cells were collected after incubation with lysis buffer (20 mmol/L Tris, 150 mmol/L NaCl, 1% Triton X‐100) containing 1 mmol/L PMSF (Beyotime Biotechnology, Shanghai, China) for 20 minutes, and cellular supernatant (total protein) was collected by centrifugation at 12000 *g* for 15 minutes at 4°C. Subsequently, 50 μL of streptavidin‐agarose beads were added to the cell supernatant and incubated for 1 hour at 4°C on a rotary mixer to bind the biotin‐labelled membranous protein. The cellular precipitation (membranous protein) was collected by centrifugation at 12000 *g* for 1 minute at 4°C, and the cellular supernatant (cytoplasmic protein) was collected into a new tube. The cellular precipitation (ie the centrifuged cellular pellet) was washed three times with cold lysis buffer and was centrifuged. The total proteins, membranous proteins and cytoplasmic proteins were analysed by Western blotting.

### 2.6 Western blotting

Streptavidin‐agarose bead–bound proteins were released in 5× loading buffer (Beyotime Biotechnology) at 55°C for 30 minutes. Total proteins and cytoplasmic proteins were boiled at 100°C for 10 minutes. Proteins were separated using a 10% SDS‐PAGE gel for 120 minutes (the amount of protein loaded per channel was 50 μg) and were then transferred to polyvinylidene‐difluoride membranes (0.45 μm pore size). An Anti‐EAAT2 antibody (Abcam) was used at 1:1000 (v/v), and a secondary antibody (Beyotime Biotechnology) was used at 1:1000 (v/v). An enhanced chemiluminescence (ECL) kit (Beyotime Biotechnology) was used for protein‐band exposure. The grey value of each protein band was quantified using ImageJ software to determine the total, membranous and cytoplasmic expression levels of EAAT2 and its mutants. The expression levels of total proteins, membranous proteins and cytoplasmic proteins were normalized to corresponding levels of actin (Beyotime Biotechnology), integrin β1 (Cell Signaling Technology), and α‐tubulin (Beyotime Biotechnology), respectively. Data were standardized and expressed as a percentage of protein expression compared with that of WT EAAT2.

### 2.7 Electrophysiology

The membrane current of a single isolated HeLa cell—transfected for 24 hours with a WT EAAT2 plasmid or mutant plasmids including R87A, R87K, R87Q, R87S, K90A, K90R, P95A and P95G EAAT2—was recorded via whole‐cell patch‐clamp electrophysiology at room temperature (21 to 23°C). Standard extracellular solution (pH 7.2) contained the following (in mmol/L): 140 NaCl, 4 KCl, 2 CaCl2.2H_2_O, 1 MgCl_2_.6H_2_O, 10 HEPES and 10 glucose. In some experiments, extracellular NaCl was equimolarly substituted with NaSCN. For determination of anion currents, cells were transferred to extracellular solutions containing sodium salts (pH 7.2) as follows (in mmol/L): 100 NaSCN, 40 NaCl, 4 KCl, 2 CaCl_2_.2H_2_O, 1 MgCl_2_.6H_2_O, 10 HEPES and 10 glucose. Patch pipettes (input resistances: 5‐7 MΩ) were filled with a standard intracellular solution containing the following (in mmol/L): 130 KCl, 5 MgCl2.6 H2O, 10 HEPES and 5 EGTA (pH 7.2). The pH was adjusted to 7.2 with 1 mol/L KOH, and osmotic pressure was set at 300 mOsmol/kg‐H_2_O with sucrose.

For recordings of currents, cells were voltage‐clamped at 0 mV for at least 1 minute between test sweeps, and voltage ramps from −100 to +100 mV in 10 mV steps with 1.5‐seconds durations over a 10‐seconds interval were applied. After establishing a stable recording of currents, to compare the membrane currents at different voltages of HeLa cells stably expressing WT EAAT2 or mutant EAAT2 in the uptake buffer containing l‐glutamate, 0.5 mmol/L of l‐glutamate was applied into the extracellular solution for 5 minutes, followed by a 3‐min washout. Data were recorded with a MultiClamp 700B (Molecular Devices, Axon Instruments), digitized at 5 kHz with Digidata 1440A (Axon Instruments), filtered at 1 kHz and analysed with Clampex 10.4 software (Axon Instruments).

### 2.8 Statistical analysis

Data are expressed as the mean ± standard deviation (SD) of three independent experiments. One‐way ANOVAs with Dunnett's T3 tests were used for determining significant differences among groups, via SPSS 21.0 software. *P* < 0.05 was considered to be statistically significant.

## 3 RESULTS

### 3.1 Effects of TM2 mutants on EAAT2 uptake function

**FIGURE 1:**
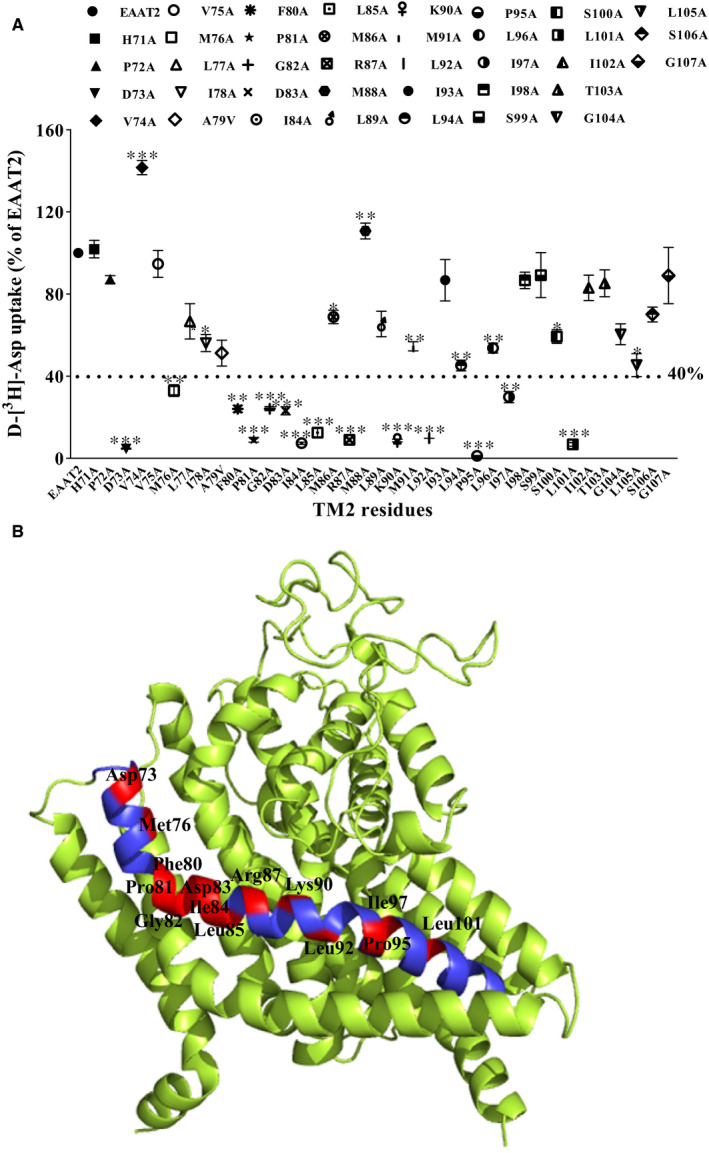
Alanine‐scanning mutations of TM2 residues in EAAT2. A, Detection of D‐[^3^H]‐aspartate uptake in WT EAAT2 and alanine‐substituted EAAT2 mutants. HeLa cells were washed twice with choline chloride solution, after which D‐[^3^H]‐aspartate uptake was measured. The experimental data were derived from three independent tests of functional activity (n = 3). The data are expressed as the percentage of D‐[^3^H]‐aspartate uptake by EAAT2 and are reported as the mean ± SD (n = 3). A one‐way ANOVA with a Dunnett T3 test was used for statistical analysis via SPSS 21.0 software. In comparison with WT EAAT2, *P < 0.05, **P < 0.01 or ***P < 0.001. B, The homologous model of EAAT2 (template: PDB; ID: 5LLU^29^; sequence identity: 63.47%) showing the 14 alanine mutants in TM2 that had reduced transporter activity by more than 60% (compared with that of WT EAAT2)

In order to identify amino acids in TM2 that critically influence EAAT2 function, we used alanine‐scanning mutations to mutate 37 residues in TM2 of EAAT2 and examined the uptake of D‐[^3^H]‐aspartate by these mutated EAAT2s. The alanine residue, Ala79, was replaced by valine—an amino acid with a comparatively larger side chain—to determine whether specific residues play important roles in EAAT2 function. Compared with that of wild‐type EAAT2 (WT EAAT2), 23 mutants maintained more than 40% of normal uptake function in EAAT‐transfected HeLa cells in vitro (Figure 1A).

The uptake functions of fourteen mutants—including D73A, M76A, F80A, P81A, G82A, D83A, I84A, L85A, R87A, K90A, L92A, P95A, I97A and L101A—were significantly decreased compared with that of WT EAAT2, at 4.70 ± 0.22%, 32.98 ± 2.83%, 24.04 ± 1.64%, 8.99 ± 1.18%, 24.20 ± 0.99%, 23.16 ± 2.00%, 6.48 ± 1.65%, 13.12 ± 1.61%, 8.98 ± 0.82%, 8.71 ± 0.63%, 9.85 ± 0.07%, 1.23 ± 0.15%, 29.81 ± 2.78% and 6.8 ± 0.90%, respectively, of that of WT EAAT2, which can be seen in Figure 1B. These results indicate that these sites on TM2 mentioned above are essential for the transport activity of EAAT2. Interestingly, we found that the transporter activity of a mutant with seven adjacent residues (Phe80, Pro81, Gly82, Asp83, Ile84, Leu85 and Arg87) substituted with alanine was significantly reduced to less than 30% of that of WT EAAT2. In addition, compared with the transport activity of WT EAAT2, the transport activities of the V74A and M88A mutants were increased (141.52 ± 2.01% and 110.64 ± 2.24%, respectively); a possible explanation for this result is that the molecular structure of these mutant proteins may be more beneficial to the substrate transport process. However, we were unable to conduct further experiments in our present study. Based on Kyte‐Doolittle hydrophobicity analysis, we found that these seven residues were located in the region with the lowest hydrophobicity in TM2 and the least hydrophilicity, whereas the entire TM2 region belonged to a larger hydrophobic domain (Figure S1). Therefore, we further studied mutants with less than 40% transport activity as compared to that of WT EAAT2, since these sites may play important roles in EAAT2.

### 3.2 Kinetic analyses of TM2 mutants with significantly reduced D‐[^3^H]‐aspartate uptake

In order to investigate whether the reduced transporter activities in TM2‐alanine‐substitution EAAT2 mutants also affected kinetic parameters of EAAT2, fourteen alanine mutants (D73A, M76A, F80A, P81A, G82A, D83A, I84A, L85A, R87A, K90A, L92A, P95A, I97A and L101A) were subjected to kinetic analyses in HeLa cells in vitro. Alanine substitutions of Leu85, Leu92 and Leu101 significantly increased Michaelis constant (K_m_) values, indicating that these binding affinities to D‐[^3^H]‐aspartate were significantly lower than that of WT EAAT2. On the contrary, after alanine substitutions of Arg87 and Pro95, both the K_m_ and maximum velocity (V_max_) values were significantly decreased, indicating that the binding affinities of these amino acid residues to D‐[^3^H]‐aspartate were significantly increased compared with that of WT EAAT2, whereas the rate of substrate transport was decreased. The normalized V_max_ values of 14 alanine mutants were significantly decreased, indicating that these amino acid residues also played an important role in the substrate turn‐over rate of EAAT2s. Among them, Asp73, Met76, Phe80, Pro81, Gly82, Asp83, Ile84, Lys90 and Ile97 only changed V_max_ values but not K_m_ values (Table 1).

**TABLE 1:** Kinetic parameters of wild‐type and mutant EAAT2

	K_m_ (μmol/L)	V_max_ (% of EAAT2)
EAAT2	27.31 ± 1.61	100
D73A	22.62 ± 5.81	0.72 ± 0.06^***^
M76A	50.71 ± 16.82	29.99 ± 5.20^**^
F80A	31.97 ± 1.36	15.57 ± 1.37^***^
P81A	21.72 ± 2.56	2.78 ± 0.31^***^
G82A	29.29 ± 3.13	13.4 ± 1.13^***^
D83A	49.89 ± 4.54	23.10 ± 0.91^***^
I84A	15.15 ± 3.57	3.82 ± 0.23^***^
L85A	162.58 ± 11.87^*^	5.80 ± 0.38^***^
R87A	10.05 ± 2.10^**^	1.65 ± 0.45^***^
K90A	36.71 ± 11.15	5.70 ± 0.74^***^
L92A	309.80 ± 23.74^*^	8.82 ± 0.57^***^
P95A	4.91 ± 2.03^**^	0.13 ± 0.03^***^
I97A	36.17 ± 3.53	21.65 ± 1.84^***^
L101A	488.25 ± 41.59^*^	8.71 ± 1.02^***^

Note

HeLa cells were incubated with different concentrations of D‐aspartate and were labelled with [^3^H] at room temperature (the final concentration of D‐[^3^H]‐aspartate was 0.4 μCi,^55^ and that of unlabelled D‐aspartic acid was 1, 3, 10, 50, 100, 500, 1000 and 1500 μmol/L). K_m_ and V_max_ values were calculated via non‐linear regression of the Hill Equation via Origin 7.5 software (Northampton, MA, USA). Each mutant V_max_ was expressed as a percentage of that of wild‐type EAAT2. The V_max_ value of WT EAAT2 was 278.85 ± 19.76 pmol/mg/min. Data are shown as the mean ± SD (n = 3). Asterisks denote statistical differences (via one‐way ANOVAs) between values for WT EAAT2 and mutants (* *P* < 0.05, ** *P* < 0.01, *** *P* < 0.001).

### 3.3 D‐[^3^H]‐aspartate uptake in additional mutants

**FIGURE 2:**
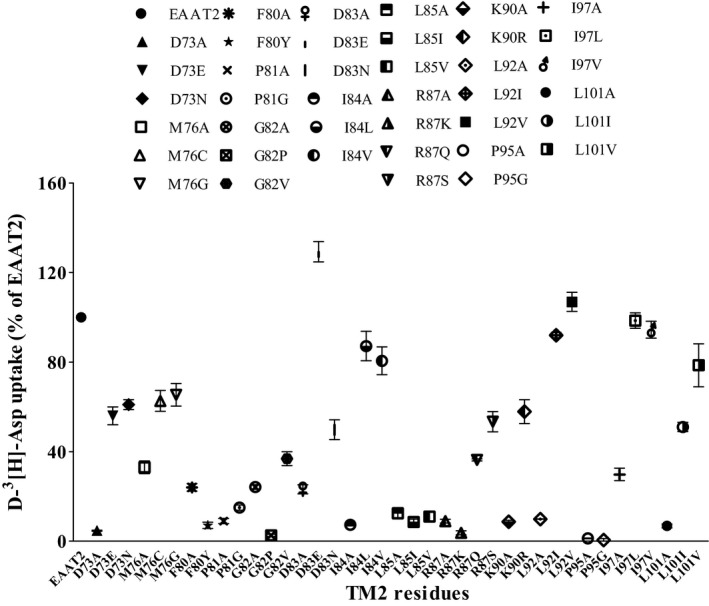
Additional mutations in functionally impaired TM2‐alanine‐substitution EAAT2 mutants. The effects of additional mutations in 14 severely impaired alanine mutants were determined. The uptake of D‐[^3^H]‐aspartate was determined in three independent experiments. Data are expressed as the percentage of D‐[^3^H]‐aspartate uptake relative to that of WT EAAT2 and are presented as the mean ± S.D (n = 3)

To further investigate whether the side‐chain structures of the critical TM2 amino acids that we discovered were necessary for EAAT2 function, we substituted these residues with similar amino acids (Figure 2).

**FIGURE 3:**
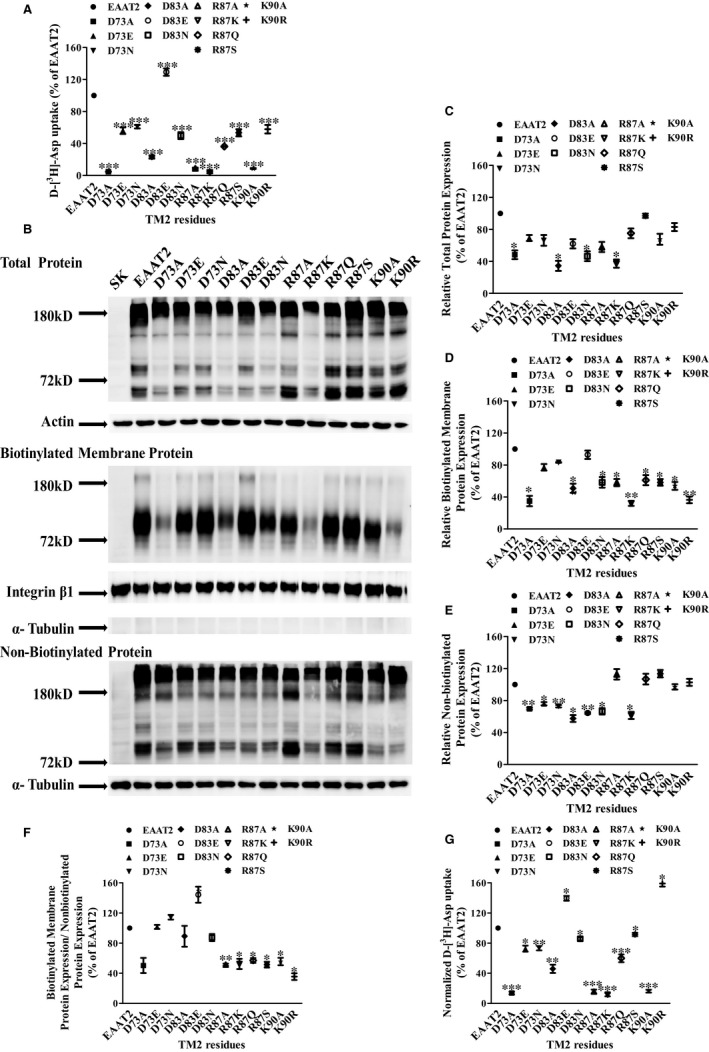
Protein expression of TM2‐alanine‐substitution mutants with significantly reduced aspartate uptake and their alternate mutations with residues classified as acidic and basic amino acids. A, The activities of alanine mutants at sites classified as acidic and basic amino acids and their alternate mutations were expressed as a percentage of the activity of WT EAAT2. B, The total protein, biotinylated membranous protein and non‐biotinylated protein of WT EAAT2 and EAAT2 mutants were determined by Western blotting. C, The total protein of each mutant was normalized by the actin level in each sample and was expressed as a percentage of that of WT EAAT2. D, The biotinylated membranous protein of mutants was normalized by the level of integrin β1 in each sample and was expressed as a percentage of that of WT EAAT2. E, The non‐biotinylated protein of the mutants was normalized by the level of α‐tubulin in each sample and was expressed as a percentage of that of WT EAAT2. F, The ratio of biotinylated membranous protein to non‐biotinylated protein of the mutants was expressed as a percentage of that of WT EAAT2. G, Standardized ratio of D‐[^3^H]‐aspartate uptake to membrane protein expression. Data are presented as the mean ± SD (n = 3). In comparison with WT EAAT2, *P < 0.05, **P < 0.01 or ***P < 0.001

Asp73 and Asp83 were replaced by glutamate, which is an acidic amino acid that contains a carboxyl group and a comparatively longer side chain than that of aspartate. In other mutants, Asp73 and Asp83 were replaced by asparagine, which is an amino acid with an amide group that is similar to that of aspartate. Both glutamate and asparagine substitutions increased the function of transport (Figures 2 and 3A). Arg87 and Lys90 were replaced by the basic amino acids, lysine and arginine, respectively; both of these amino acids contain nitrogenous base groups. D‐[^3^H]‐aspartate uptake was increased in the K90R mutant but was decreased in the R87K mutant (Figures 2 and 3A). Therefore, Arg87 was next replaced with glutamine and serine to determine whether these substitutions could improve transporter function. We found that D‐[^3^H]‐aspartate uptake in both R87Q and R87S mutants was increased (Figures 2 and 3A).

**FIGURE 4:**
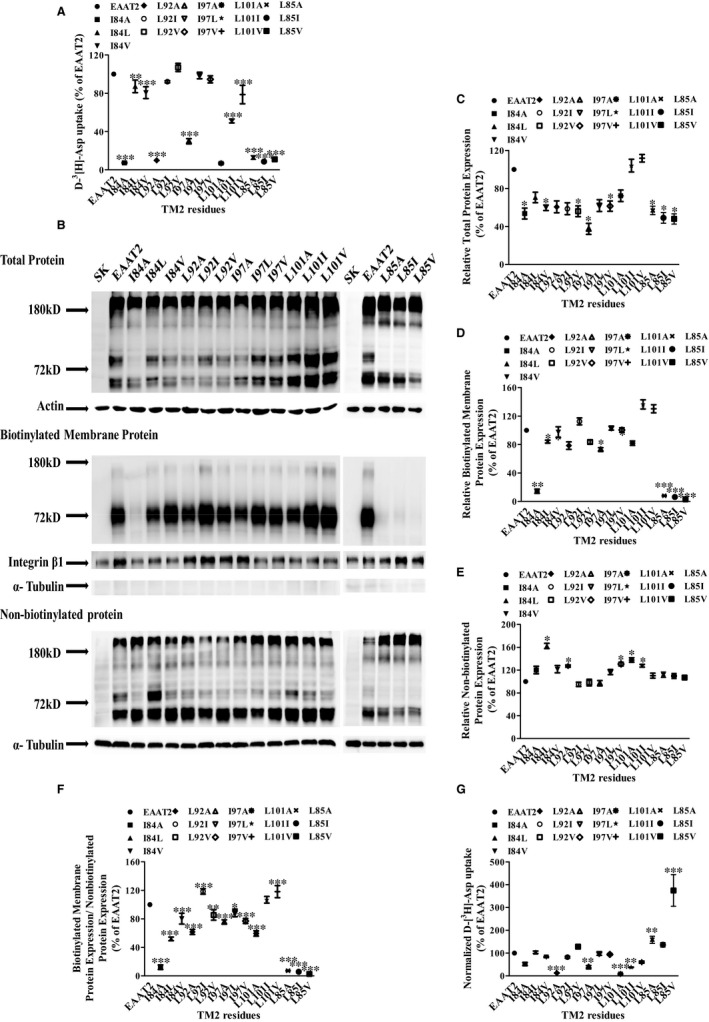
EAAT2 protein expression of TM2‐alanine‐substitution mutants with significantly reduced aspartate uptake and their alternate mutants with residues classified by different side‐chain lengths. A, The activities of alanine mutants classified by side‐chain structures and their alternate mutations, expressed as a percentage of that of WT EAAT2. B, Western blotting of EAAT2 total protein, biotinylated membranous protein and non‐biotinylated protein were probed with anti‐EAAT2. (C‐E) Densitometric analyses of the EAAT2 total protein, biotinylated membranous protein and non‐biotinylated protein via ImageJ. F, The ratio of EAAT2 biotinylated membranous protein expression to non‐biotinylated protein expression in each mutant was expressed as a percentage of that of WT EAAT2. G, D‐[^3^H]‐aspartate uptake normalized to relative cell‐surface expression as a percentage of that of WT EAAT2. Data are shown as the mean ± SD (n = 3). In comparison with WT EAAT2, *P < 0.05, **P < 0.01 or ***P < 0.001

The substitution of hydrophobic residues (Ile84, Ile97, Leu85, Leu92 and Leu101) with similar amino acids containing similar side‐chain structures restored the D‐[^3^H]‐aspartate uptake of all mutants except for the L85I and L85V mutants in EAAT2, indicating that leucine plays an irreplaceable role at site 85 (Figures 2 and 4A).

**FIGURE 5:**
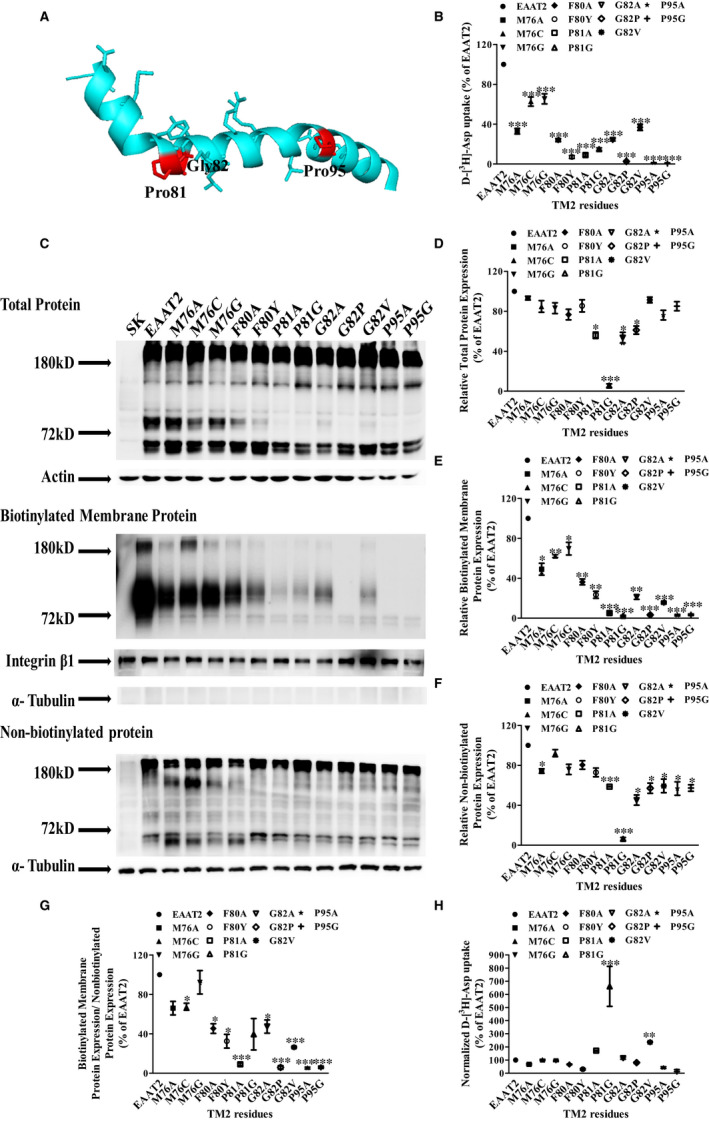
Protein expression of TM2‐alanine‐substitution mutants with significantly reduced aspartate uptake and their alternate mutants in the rest of the residues. A, The distribution of three critical residues—including Pro81, Gly82 and Pro95 (highlighted in red)—in TM2 using an EAAT2‐homologous model (template: PDB; ID: 5LLU^29^; sequence identity: 63.47%). B, The activities of alanine mutants and their alternate mutations at sites in the rest of the 14 amino acids were expressed as a percentage of that of WT EAAT2. C, Protein expression of the total protein, biotinylated membranous protein and non‐biotinylated protein of EAAT2. (D‐F) Total protein, biotinylated membranous protein and non‐biotinylated protein expression of WT EAAT2 and EAAT2 mutants via Western blotting with an anti‐EAAT2 antibody. The grey value of each band was quantitatively determined via ImageJ. G, Relative biotinylated membranous protein expression of each mutant is expressed as a percentage of that of WT EAAT2. H, Normalized D‐[^3^H]‐aspartate uptake of each mutant is expressed as a percentage of that of WT EAAT2. Data are shown as the mean ± SD (n = 3). In comparison with WT EAAT2, *P < 0.05, **P < 0.01 or ***P < 0.001

The three critical residues—namely Pro81, Gly82 and Pro95—in TM2 were located at two corners of the TM2 region (Figure 5A). Pro81 and Pro95 were substituted by glycine, which has a similar ‘kink structure’ to that of proline but is more flexible. We found that the transporter activities of P81G and P95G did not significantly recover following glycine substitutions (Figures 2 and 5B), indicating that proline plays a critical role in sites 81 and 95. Gly82 was substituted with proline, which can also contribute to the formation of a ‘kink structure’ similar to that of glycine but has a larger side group. As a result of this substitution, the transporter activity of the G82P mutant was further decreased (Figures 2 and 5B). In addition, as glycine has a small side chain (hydrogen atom), substituting glycine with alanine containing a methyl side group may prevent substrate transport. Therefore, glycine was substituted with valine—which has a side chain that is slightly larger than that of alanine—to observe whether the side chain size of Gly82 contributes to transporter activity (Figures 2 and 5B). We found that there was no significant improvement in the transporter activity in the G82V mutant following valine substitutions (Figures 2 and 5B). Met76 was then replaced by glycine, which is also a non‐polar amino acid and is hydrophobic. In addition, as methionine contains a thioether group, it was substituted with cysteine containing a sulfhydryl group to determine whether sulphur (S) atoms affect transport activity of EAAT2. We found that the transporter activity of M76C and M76G mutants recovered following these substitutions (Figures 2 and 5B). Phe80 was replaced with tyrosine with the same aromatic groups, which contains hydroxyl groups and has a comparatively larger side chain; this substitution decreased transporter activity (Figures 2 and 5B). These results indicate that Phe80 had an irreplaceable effect on the transport activity of EAAT2.

### 3.4 Conservative replacement of amino acids is important for EAAT2 function

Conservative replacement of amino acids not only affects the transporter activity of EAAT2, but also affects its protein expression. EAAT2, as a membranous protein, can only exhibit normal functionality as a transporter when it is properly localized in the plasma membrane. Therefore, in order to explore whether the altered transporter functions of our mutants were related to membrane‐bound protein expression, we conducted cell‐surface biotinylation assays for verification after performing tests on uptake activities. For this purpose, we classified our 14 critical amino acids into three types: (a) acidic and alkaline amino acids; (b) amino acids with similar side‐chain properties; and (c) all other amino acids that were not classified into the first two types. The ratio of biotinylated membranous protein expression to that of non‐biotinylated protein expression (ie relative biotinylated membranous protein expression) and the ratio of D‐[^3^H]‐aspartate uptake activity to membranous protein expression (ie normalized D‐[^3^H]‐aspartate uptake) were used to quantify membranous protein expression and transporter activity, respectively, of EAAT2.

Firstly, protein expression of acidic and basic amino acids in EAAT2 TM2 was detected by membrane impermeable biotin labelling and immunoblotting (Figure 3B). We showed that compared with that in WT EAAT2, the total EAAT2 protein was reduced in D73A, D83A, D83N and R87K mutants (Figure 3C), the membranous proteins of D73A, D83A, D83N, R87A, R87K, R87Q, R87S, K90A and K90R mutants were reduced (Figure 3D), and the cytoplasmic proteins of D73A, D73E, D73N, D83A, D83E, D83N and R87K mutants were reduced (Figure 3E), but not in any other mutants. Relative biotinylated membranous protein expression was decreased in Arg87 and Lys90 mutants, whereas there was no change in this parameter in any other mutants (Figure 3F). In addition, normalized D‐[^3^H]‐aspartate uptake was decreased in D73A, D83A, R87A and K90A mutants. After additional mutations, we found that the transporter activity recovered significantly in all mutants except for the R87K mutant (Figure 3G). These results showed that the decreased normalized D‐[^3^H]‐aspartate uptake of D73A, D83A, R87A and K90A mutants may affect the trafficking of EAAT2 to the plasma membrane. Importantly, compared with that of WT EAAT2, the transport activity of mutant D83E was increased by more than 29.33 ± 4.58%, and the transport activity obtained after normalization of the corresponding membrane‐bound protein expression level was increased by 39.51 ± 3.25% (Figure 3A,G).

Importantly, compared with WT EAAT2, the transport activity of mutant D83E increased by more than 29.33 ± 4.58%, and the transport activity obtained after normalization of the corresponding membrane‐bound protein expression level increased by 39.51 ± 3.25% (Figure 3A,G).

Secondly, protein expression of amino acids with similar side‐chain properties to those of EAAT2 TM2 was detected by membrane impermeable biotin labelling and immunoblotting (Figure 4B). We showed that the total EAAT2 proteins in all mutants (except L101I and L101V mutants) were decreased compared with those of WT EAAT2 (Figure 4C), the membranous proteins of I84A, I84L, I97A, L85A, L85I and L85V mutants were reduced (Figure 4D), and the cytoplasmic proteins of I84L, L92A, I97V, L101A and L101I mutants were increased (Figure 4E), but not in any other mutants. Relative biotinylated membranous EAAT2 protein expression levels were significantly decreased in I84A, L85A, L85I and L85V mutants, whereas these levels were significantly increased in I84V, L92I, L92V, I97A, I97L and I97V mutants compared with that in WT EAAT2; these levels in all other mutants were not significantly different from those of WE EAAT2 (Figure 4F). In addition, compared with that of EAAT2, normalized D‐[^3^H]‐Asp uptake was decreased in L92A, I97A and L101A mutants but was not significantly different in any other mutants (Figure 4G). These results indicated that significant decreases in membranous protein expression were the main reason for the decreased aspartate uptake in L85A, L85I and L85V mutants. However, I97A, I97L and I97V mutants exhibit defects in transport activity rather than plasma‐membrane targeting. Both membranous protein expression and transporter activity were significantly increased in Ile84, Leu92 and Leu101 mutants compared with that of WT EAAT2, all of which recovered following alternate mutations in these mutants. Above all, these critical amino acid residues with similar properties in terms of the side chains of EAAT2 TM2 mainly affected uptake function by altering membranous protein expression and/or transporter activity of EAAT2.

Finally, protein expression for all other amino acids from EAAT2 TM2 was detected by membrane impermeable biotin labelling and immunoblotting (Figure 5C). We revealed that the total EAAT proteins of P81A, P81G, G82A and G82P mutants were decreased compared with that of WT EAAT2 (Figure 5D). Compared with that of WT EAAT2, there were no significant differences in the total EAAT proteins in any of the other mutants (Figure 5D). The membranous proteins of M76A, M76C, M76G, F80A, F80Y, P81A, P81G, G82A, G82V, P95A and P95G mutants were reduced (Figure 5E), and the cytoplasmic proteins of M76A, P81A, P81G, G82A, G82V, P95A and P95G mutants were reduced (Figure 5F), but not in any other mutants. The relative biotinylated membranous protein expression levels of M76A, F80A, P81A, G82A and P95A—as well as those of their alternate mutants—were decreased compared with that of WT EAAT2 (Figure 5G). Normalized D‐[^3^H]‐aspartate uptake in both P81G and G82V mutants was increased, but there were no significant changes in this parameter for any of the other mutants compared with that of WT EAAT2 (Figure 5H). Among them, P81G and G82V exhibited significantly increased normalized D‐[^3^H]‐aspartate uptake via introducing glycine and valine (similar amino acids), respectively, but these substitutions were still not sufficient to significantly increase the uptake function of these mutants. Therefore, we conclude that the uptake function of these critical amino acids was related to the expression of relative biotinylated membranous EAAT2.

### 3.5 Fourteen alanine mutants affect EAAT2 plasma‐membrane protein expression and/or interactions with substrates

**FIGURE 6:**
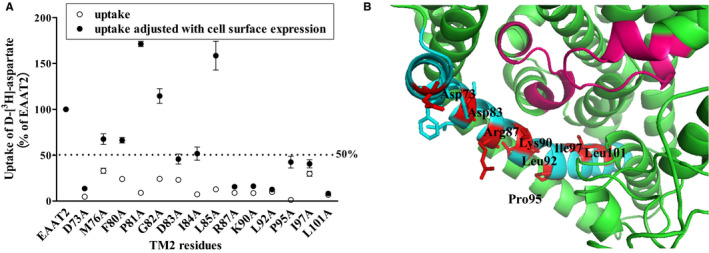
D‐[^3^H]‐aspartate uptake of different EAAT2 mutants normalized with their corresponding membrane‐bound protein expression levels of EAAT2. A, The transporter activities of fourteen alanine mutants with significantly decreased transporter activities (open circles) and the ratio of D‐[^3^H]‐aspartate uptake to membranous EAAT2 protein expression, relative to those of WT EAAT2 (set at 100; filled circles). B, The homologous model of EAAT2 (template: PDB; ID: 5LLU^29^; sequence identity: 63.47%) showing the eight alanine mutants in TM2 that had reduced transporter activity by more than 50% (compared with that of WT EAAT2) after normalization to membranous protein expression of EAAT2. TM2 sections are shown in blue, the eight mutation sites are shown in red, and HP1 and HP2 are shown in pink

In order to investigate whether the effect of the 14 alanine mutants on uptake function was caused by decreased protein levels in the plasma membrane, D‐[^3^H]‐aspartate uptake of these alanine mutants was normalized to membranous protein expression and normalized uptake activities were then compared (Figure 6A). The results showed that these normalized uptake activities of M76A, F80A, P81A, G82A, I84A and L85A recovered to more than 50% of that of WT EAAT2, suggesting that the decreased uptake function of these residues may have been affected by reduced localization of transporter expression in the plasma membrane. In addition, the normalized transporter activities of the other eight mutants, namely D73A, D83A, R87A, K90A, L92V, P95A, I97A and L101A, were still decreased by more than 50%, suggesting that the substitution of these residues may change the interaction between the transporter and its substrate. In particular, the transport activities obtained after normalization of the expression levels of the corresponding membrane‐bound proteins of mutants P81A, G82A and L85A were increased significantly (171.24 ± 2.79, 114.54 ± 8.01, 158.41 ± 15.68, respectively).

### 3.6 P95A modifies the time and voltage dependence of EAAT2‐associated currents in HeLa cells

The EAAT1 TM2 region has been found to have some mutations— such as R90L, Q93A and P98G—that alter anion permeation properties.^34^ We found that these amino acid sites corresponded to Arg87, Lys90 and Pro95 in the EAAT2 TM2 region. The uptake function and protein expression of R87A, R87K, R87Q, R87S, K90A, K90R, P95A and P95G EAAT2 were determined by the above e experiments (Figures 2, 3 and 5). Hence, we further investigated the relationship between the mutation of these sites and the function of EAAT2‐related anion currents. In order to further determine whether the locus of alanine and alternate mutations in EAAT2 TM2 was also involved in anion currents, we expressed WT EAAT2, R87A, R87K, R87Q, R87S, K90A, K90R, P95A and P95G mutants transiently in HeLa cells and performed whole‐cell patch‐clamp experiments to measure EAAT2‐associated currents. A potassium chloride (KCl)–based solution was used intracellularly as a dialysis fluid, which allowed for the glutamic acid absorption cycle of all physical transportation. We used Cl^‐^ as the main intracellular and extracellular anion in order to increase EAAT2‐associated anion currents, so that anion currents of EAAT2 were significantly higher than that of uptake currents and intrinsic currents; this manipulation allowed us to measure the anion currents of EAAT2 independently, specifically by comparing alterations of such currents in HeLa cells transfected with EAAT2‐specific mutants relative to those in HeLa cells transfected with WT EAAT2.

**FIGURE 7:**
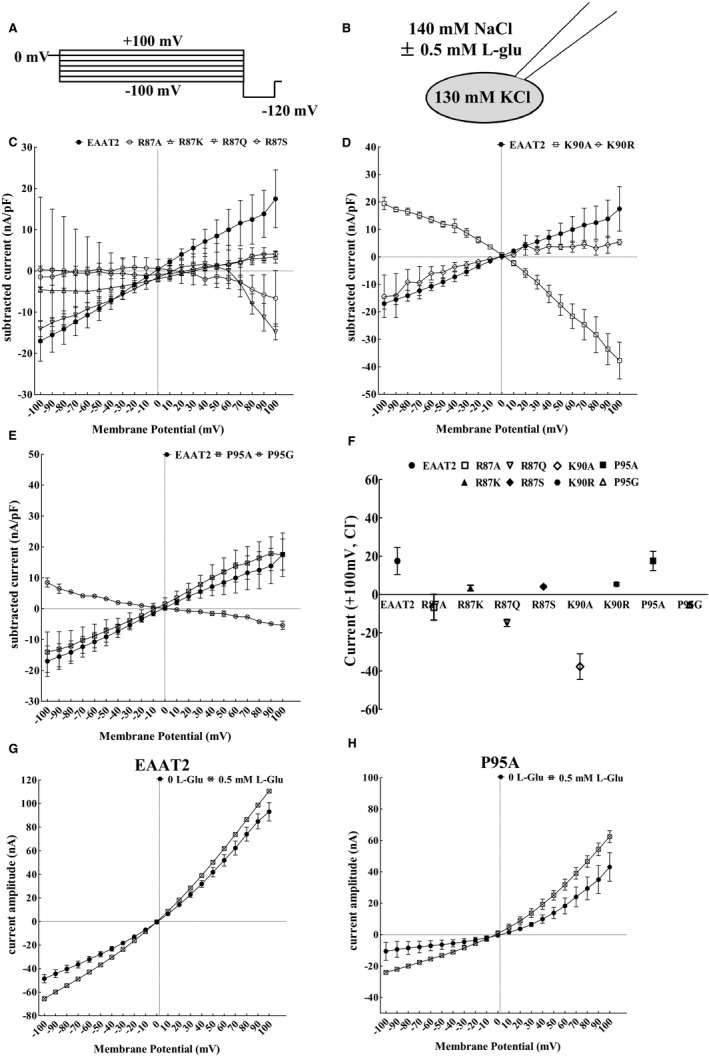
P95A affects EAAT2‐associated currents in HeLa cells. A, Representative whole‐cell currents from HeLa cells expressing WT EAAT2 or EAAT2 mutants were held at 0 mV, and voltage steps between −100 mV and +100 mV were followed by a fixed step to −120 mV. B, Patched cells were dialysed with 130 mmol/L intracellular KCl‐based solution and perfused with 140 mmol/L extracellular NaCl‐based solution in the absence or presence of 0.5 mmol/L L‐glutamate. (C‐E) Current‐voltage relationships from HeLa cells transiently expressing WT EAAT2 or EAAT2 mutations in Arg87 (C), Lys90 (D) and Pro95 (E) are shown as the subtracted values of current amplitudes induced by 0.5 mmol/L L‐glutamic acid or the absence of L‐glutamic acid at different membrane potentials. F, The subtracted values of current amplitudes elicited by 0.5 mmol/L L‐glutamic acid of the absence of l‐glutamic acid were measured at +100 mV in HeLa cells expressing either WT EAAT2 or mutants with R87A, R87K, R87Q, K90A, K90R, P95A or P95G point mutations. G, Current‐voltage relationships of the instantaneous currents of HeLa cells expressing WT EAAT2 in the absence and presence of 0.5 mmol/L l‐glutamic acid. H, Current‐voltage relationships of the instantaneous currents of HeLa cells transfected with P95A EAAT2 in the absence and presence of 0.5 mmol/L l‐glutamate. Data are presented as the mean ± SD of absolute current data from three cells per experimental group for all figures. In comparison with WT EAAT2, *P < 0.05, **P < 0.01 or ***P < 0.001

Figure 7A,B shows the responses of whole‐cell currents to typical voltage steps between −100 mV and +100 mV in HeLa cells with transient transfection of WT EAAT2 or various EAAT2 mutants, as well as responses to fixed voltage steps to 120 mV. These responses were represented by the subtracted values of current amplitudes between 0.5 mmol/L l‐glutamic acid and non–l‐glutamic acid (Figure 7C–E). The current subtraction between 0.5 mmol/L l‐glutamic acid and non–l‐glutamic acid in P95A EAAT2 was similar to that of WT EAAT2 at 100 mV (Figure 7G,H). In contrast, the EAAT2‐associated currents of R87K, R87S and K90R mutants were not significantly different in the absence and presence of l‐glutamic acid (Figure 7F). Our results suggest that P95A EAAT2 caused a significant change in the time and voltage dependence of EAAT2 currents after addition of 0.5 mmol/L l‐glutamic acid, whereas the currents of R87K, R87S and K90R mutants did not show this dependence. In addition, with the addition of substrates, the current amplitudes in the R87A, R87Q and K90A mutants are greatly reduced, which makes it impossible to further study such currents in these mutants.

### 3.7 P95A decreases EAAT2‐associated anion currents in HeLa cells

**FIGURE 8:**
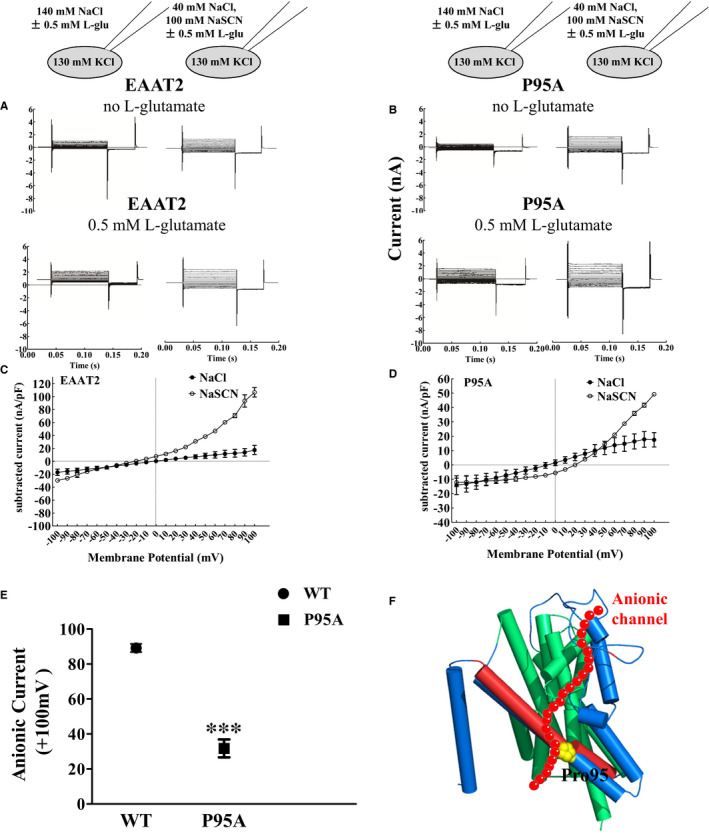
P95A decreases EAAT2‐associated anionic currents. (A and B) Representative whole‐cell current traces recorded from HeLa cells transiently transfected with EAAT2 transporters. Cells were dialysed with a KCl‐based intracellular solution and perfused either in the presence or absence of 0.5 mmol/L l‐glutamic acid as an extracellular solution containing Cl‐ or SCN‐. Cells were held at 0 mV, and voltage steps between −100 and +100 mV at −120 mV intervals were applied in WT EAAT2 (A) and P95A EAAT2 (B). (C and D) Voltage dependence of isochronal current amplitudes of different anions composed of Cl‐ and SCN‐ in WT EAAT2 (C) and P95A EAAT2 (D), shown as the subtracted values of current amplitudes elicited in the presence or absence of 0.5 mmol/L l‐glutamic acid. E, The subtracted values of the current amplitudes in the extracellular solution containing SCN‐ and Cl‐, expressed as the anionic currents from WT EAAT2 and P95A EAAT2. Here, the current amplitudes represented the subtraction of currents between those in the presence and absence of 0.5 mmol/L l‐glutamic acid measured at +100 mV. F, Localization of proline 95 in the EAAT2 homologous topology model is shown (template: PDB; ID: 5LLU^29^; sequence identity: 63.47%), which may take part in the anionic channel (denoted by the red ball chain). Data are presented as the mean ± SD of absolute current data from three cells per group for all figures. In comparison with WT EAAT2, *P < 0.05, **P < 0.01 or ***P < 0.001

We found that the P95A mutant affected EAAT2‐related currents in HeLa cells. Glutamate and Na^+^ are necessary for the gating of anionic channels, and the directionality of anionic fluxes passing through EAAT2 is independent of the transport process. In addition to its main physiological anion, Cl^‐^, EAAT2 can permeate other monovalent anions, and the relative order of such permeabilities is as follows: SCN^−^>CLO4^−^ >NO^−^> I^−^>Br^−^>Cl^−^>F^−^. Therefore, in order to further explore whether this mutation specifically affects EAAT2‐associated anion currents, 100 mmol/L thiocyanate (SCN^‐^) was used instead of partial Cl^‐^ in the extracellular solution. By substituting partial Cl‐ for SCN‐ in extracellular fluid to increase the anionic permeability of EAAT2, the current subtraction between the presence and absence of l‐glutamic acid was measured and compared with that in extracellular fluid containing 140 mmol/L NaCl. As shown in Figure 8A,B, the whole‐cell currents in HeLa cells transiently transfected with WT EAAT2 and the P95A mutant were measured in two kinds of extracellular fluids (NaCl and NaSCN) in the absence and presence of 0.5 mmol/L l‐glutamate. We found that after an SCN^‐^ solution replaced a partial Cl^‐^ solution, the current subtractions between the presence and absence of l‐glutamic acid in WT EAAT2 and P95A EAAT2 were higher than they were in 140 mmol/L NaCl (Figure 8C,D). The current subtraction between SCN^‐^ substitution and before substitution (in NaCl) was used to reflect changes in EAAT2‐related anion currents. We found that the anion currents of the P95A mutant were significantly lower than those of WT EAAT2 (Figure 8E). Therefore, we concluded that the P95A mutant decreased EAAT2‐related anion currents (Figure 8F).

## 4 DISCUSSION

TM2s of EAATs are involved in forming the trimer interfaces of EAATs; the movement of TM2 in EAATs remains relatively unchanged relative to the trimer interface during substrate transport,^50^ which is a highly conserved property among transporter subtypes and plays an important role in maintaining transporter balance. However, research on the structure‐function relationship of TM2 in EAATs has been limited. In the present study, the effect of TM2 on the normal function of EAAT2 was investigated via alanine‐scanning mutations. We found that alanine substitutions of 14 TM2 residues reduced D‐[^3^H]‐aspartate uptake by EAATs by more than 60%. After the cell‐surface protein levels of EAATs were standardized across groups, D‐[^3^H]‐aspartate uptake remained significantly decreased by >50% in eight alanine mutants (Figure 6A). Notably, the normalized transport activity of mutants P81A and L85A was significantly increased (specific values) compared with that of WT EAAT2. It was found that in the central region of the trimer, TM2 and TM5 passed through the membrane at an oblique angle to form the thinnest part of the transporter and to separate the extracellular fluid and cytoplasm through ~15 Å.^51^ As shown in Figure 6B, except for Pro95, the other seven residues faced the internal channels formed by different transmembrane domains and were located near HP1 and HP2. It has previously been reported that in Glt_ph_, Val364 of HP2 and Phe55 of TM2 (EAAT2 Pro81), as well as Val420 of HP2 and R61 (EAAT2 Arg87) or Lys64 (EAAT2 Lys90) of TM2, become closer to the cytoplasmic conformation of EAAT2 and that TM2 has a significant interaction with HP2.^19^ EAAT2 appears in the inward‐facing conformation through three different conformational changes in Glt_Ph_ and two changes in EAAT_cryst_. The substrate‐binding zone is next to TM2, which is close to HP2 and away from HP1 (Figure S2A–E). In addition, although the Pro98 site in EAAT1 (Pro95 site in EAAT2) is unable to enter the aqueous environment, proline introduces a ‘kink’ structure in the helix and limits conformational changes, thus affecting the flexibility of the α‐helix and the penetration of anions, as well as the conformational changes required to open the calcium channel.^34^ Additionally, we found that the K_m_ value of Pro95 was significantly lower than that of WT EAAT2. Compared with that of WT EAAT2, the transport activities of P81A, G82A and L85A mutants were reduced by more than 50%, but the transport activities obtained after normalization of the corresponding membrane‐bound protein expression levels were significantly increased (Figure 6A). These results may be explained by the fact that the alanine mutations may have induced EAAT2 to incur obstacles in the process of localization to the plasma membrane, but that the molecular structure of the mutant proteins may have been more beneficial for substrate transport. Nevertheless, the specific mechanism requires further elucidation. These results suggest that these sites might be involved in the formation of substrate transport pathways of EAAT2.

Via sequence alignment, we found that Gly82, Arg87 and Pro95 were highly conserved in nine types of the analysed transporters; Phe80, Pro81, Leu85 and Leu101 were conserved in eight transporters; Lys90, Leu92 and Ile97 were conserved in five transporters; and Ile84 was conserved in two transporters. Furthermore, Asp73, Met76 and Asp83 appeared to be unique to EAAT2 (Figure S2F). We found that most highly conserved amino acids were nonpolar and hydrophobic. The exceptions included Asp73 and Asp83 (acidic amino acids) and Arg87 (basic amino acid). Our hydrophobicity analysis also showed that the whole TM2 region belonged to a relatively hydrophobic environment. Therefore, the hydrophobicity of the TM2 region might be important for the transporter function of EAATs. In addition, we found that the seven adjacent key residues of Phe80, Pro81, Gly82, Asp83, Ile84, Leu85 and R87A in the middle and upper segments of TM2 exhibited weak hydrophilicities; hence, we hypothesize that these residues may be exposed to aqueous environments and mediate the transport of substrates (Figure S1).

We found that four critical amino acids with acid/basic properties—including Asp73, Asp83, Arg87 and Lys90—may be involved in the formation of the substrate transport pathway of EAAT2. These four key amino acids affected normalized D‐[^3^H]‐aspartate uptake (Figure 3G) but not the expression of relative biotinylated membranous protein (Figure 3F). Glutamate and asparagine substitutions of Asp73 each introduced a hydrophilic carboxyl group and amide group, which may promote the recognition of substrates and thus enhance the transport activity of membranous EAAT2. However, glutamate substitution of Asp83 restored uptake function above that of normal levels, whereas asparagine substitution only partially recovered uptake function. The side chain of glutamate is longer than that of aspartic acid and the hydrophilicity of its carboxyl group is larger than that of its amide group, which may make the structure of the transporter more conducive to the binding and transport of the substrate. Therefore, the length of the side chain at site 83 and the hydrophilicity of the carboxyl group at this site may play an important role in the transport function of EAAT2. In the present study, we showed that the transport function of Arg87 was restored after it was substituted by serine. Some previous simulation studies have shown that serine‐rich rings are of great importance in substrate recognition and binding and, in particular, its hydroxyl side chain is necessary for substrate and cation selectivity.^52^, ^53^ Therefore, we speculated that the serine in site 87 may have an important effect on the transport function of EAAT2. However, the hydrophilicity of the amide bond of glutamine is weaker than that of the hydroxyl group, so the recovery of transporter activity via glutamine substitution was less than that in the serine substitution. The side chain of arginine is composed of a 3‐C aliphatic straight chain, its distal end is capped by a guanidine group, and its pKa is 12.48; as such, arginine is always protonated and positively charged under physiological pH. Because of the conjugation between the double bond and the lone nitrogen pair, the positive charge is delocalized, making it possible to form multiple hydrogen bonds. Previous studies have shown that hydrogen bonds play an important role in the stability of multiple protein subunits.^54^ In our present study, we found that the transporter activity of the R87K mutant was further decreased. Therefore, the formation of arginine at site 87 may be critical for the transporter activity of EAAT2. It has been reported that Arg87 can enter the aqueous environment,^36^ which is close to the residue on HP2 in the inward‐facing conformation.^19^ We found that Arg87 was close to the substrate‐binding site and protruded at the interface between transport and trimer domains (Figure 6B). In addition, we found that the K_m_ value of the R87A mutant was significantly lower than that of WT EAAT2. These results suggest that Arg87 might be associated with substrate binding and co‐ordinate substrate transport by interacting with other nearby residues. Our results showed that when Lys90 was replaced by arginine, the transporter activity of normalized membranous EAAT2 recovered and was even enhanced relative to that of WT EAAT2. It has been shown that EAAT2 Lys90, like Arg87, is closer to HP2 in the inward‐facing conformation.^19^ EAAT1 Q93A (EAAT2 K90A) has been shown to induce significant changes in the relative anionic permeabilities of the activation conductance of D‐aspartic acid and l‐glutamic acid compared with those of WT EAAT1.^34^ Hence, it is possible that the positive charge of Lys90 may play an important role in the anionic selectivity of the uncoupled conductance of EAAT2, thus facilitating the transport of substrates.

For the five crucial amino acids with similar side‐chain properties to those of WT EAAT2 residues, except for Leu85, the normalized D‐[^3^H]‐aspartate uptake of the other four mutants (Ile84, Leu92, Ile97 and Leu101) was significantly restored after being replaced by amino acids with similar side‐chain structures (Figure 4G). These findings suggest that the hydrophilicities and side‐chain structures of these residues may be critical to the transporter activity of EAAT2. On the contrary, when Leu85 was substituted by alanine, isoleucine or valine, the relative EAAT2 biotinylated membranous protein was significantly reduced, suggesting that leucine at site 85 in TM2 may have irreplaceable steric effects and an important side‐chain length. In addition, we found that the K_m_ values of L85A, L92A and L101A mutants were significantly higher than those of WT EAAT2 (Table 1), suggesting that these residues may affect the binding affinity of EAAT2 to substrates.

For five critical amino acids out of the remaining amino acids, EAAT2 proteins in Met76, Phe80, Pro81, Gly82 and Pro95 mutants remained in the cytoplasm after alanine‐scanning mutations (Figure 5F), suggesting that these amino acids may affect the trafficking of EAAT2 to the plasma membrane (Figure 5E). Mutations in M76A, F80A, P81A, G82A and P95A and their alternative mutants affected the expression of relative biotinylated membranous EAAT2 protein (Figure 5G) but did not affect normalized D‐[^3^H]‐aspartate uptake (Figure 5H). It is known that methionine and glycine are both non‐polar amino acids. Additionally, glycine is the only non‐chiral amino acid and is also small and flexible. The membranous protein expression of EAAT2 was increased after the substitution of glycine at site 76, thus enhancing EAAT2 uptake function. Therefore, site 76 may require some structural flexibility for proper spatial expression of EAAT2. However, the uptake functions following alternative mutations of F80Y, P81G, G82P and P95G mutants were decreased significantly compared with that of site 76. Both EAAT2 membranous protein expression and transporter activity of the F80Y mutant were significantly decreased compared with that of WT EAAT2, suggesting that phenylalanine at site 80 in TM2 may play an irreplaceable role in EAAT2. Moreover, our study demonstrated that the P95A mutant decreased EAAT2‐associated anion currents in HeLa cells (Figure 8E). We speculate that the decreased transporter activity in the P95A mutant putatively led to a decrease in ionic concentration gradients and/or the properties of the anionic channel may have changed to decrease ionic conductance of anion currents specifically. This phenotype may have led to decreased anion currents in the P95A mutant compared with that of WT EAAT2. However, the EAAT2‐related currents in HeLa cells were determined by the electrophysiological experiments, under the condition that the ionic concentration gradients in the cell were constant. Hence, we speculated that the substitution of alanine at site 95 changed the properties of the anionic channel and decreased the anion currents of EAAT2. Additionally, we found that the P95G mutant did not induce EAAT2‐associated anion currents (Figure 7E). Although glycine and alanine are both nonpolar amino acids and their structures are similar, glycine has a ‘kink structure’ similar to that of proline, and it is known that the ‘kink structure’ of site 98 in EAAT1 (site 95 in EAAT2) is beneficial to the penetration of anions.^33^ Therefore, our present study showed that P95A EAAT2 was involved in mediating the opening of anionic channels and that the ‘kink structure’ in site 95 may be important to EAAT2‐associated anion currents. In addition, after Pro81 and Gly82 were replaced by glycine and valine, respectively, normalized D‐[^3^H]‐Asp uptake was enhanced after substitution, but it was not sufficient to restore the uptake function of EAAT2. Also, Pro81, Gly82 and Pro95 were located at two corners of the TM2 region (Figure 5A). Taken together, we hypothesize that the ‘kink structure’ of proline and glycine at sites 81, 82 and 95 play an irreplaceable role in the uptake function of EAAT2 and that the proline at site 95 may be related to anionic channels in EAAT2 TM2 (Figure 8F).

In conclusion, our present study revealed that the TM2 of EAAT2 is important for localization of EAAT2 to the plasma membrane, as well as subsequent substrate binding, substrate transport and conductance of anion currents. Additionally, we determined the protein transport activities, expression levels and kinetic parameters of the 14 key amino acid residues of TM2 in EAAT2 after alanine‐scanning mutations (Table 2). Additionally, our results demonstrated that changes in structural properties via site‐specific mutations in EAAT2 TM2 were related to changes in properties of EAAT2‐associated anionic channels. Structural and functional changes in the critical residues of TM2 elucidated in the present study may be involved in the pathophysiology of some neurodegenerative diseases, which may provide a structural basis for further understanding the mechanisms of such diseases.

**TABLE 2:** Protein transport activity and expression of alanine‐scanning mutations of TM2 14 crucial amino acid residues in EAAT2

Amino acid classification	Acid and alkaline amino acids				Amino acid similar to side chain properties					Amino acids of the rest				
Mutants	D73A	D83A	R87A	K90A	I84A	L85A	L92A	I97A	L101A	M76A	F80A	P81A	G82A	P95A
D‐[^3^H]‐Asp uptake	−	−	−	−	−	−	−	−	−	−	−	−	−	−
K_m_	N	N	N	N	N	+	−	N	+	N	N	N	+	−
V_max_	−	−	−	−	−	−	−	−	−	−	−	−	−	−
Biotinylated/nonbiotinylated protein	−	−	−	−	+	+	+	−	+	+	+	+	+	+
Normalized D‐[^3^H]‐Asp uptake	+	+	+	+	+	−	+	+	+	−	−	−	−	−

Note

Compared to WT EAAT2, ‘+’/‘−’ represents statistical significance and increase / decrease, and ‘N’ represents no statistical significance.

## CONFLICT OF INTEREST

The authors declare that there were no conflicts of interest related to the present study.

## AUTHOR CONTRIBUTIONS

**Dongmei Mai:** Writing‐original draft (lead). **Rongqing Chen:** Writing‐review & editing (supporting). **Ji Wang:** Project administration (equal). **Jiawei Zheng:** Data curation (equal); Software (equal). **Xiuping Zhang:** Project administration (equal). **Shaogang Qu:** Funding acquisition (lead); Writing‐review & editing (lead).

## Supporting information

Fig S1‐S2
