# Supplementary material for: Critical amino acids in the TM2 of EAAT2 are essential for membrane‐bound localization, substrate binding, transporter function and anion currents
Source: J Cell Mol Med. 2021 Feb 1;25(5):2530–48. doi: 10.1111/jcmm.16212 (PMC7933967; doi:10.1111/jcmm.16212)
Supplement: Supplementary file 1 — Fig S1‐S2 [file JCMM-25-2530-s001.docx]

**SUPPLEMENTARY MATERIALS**

**
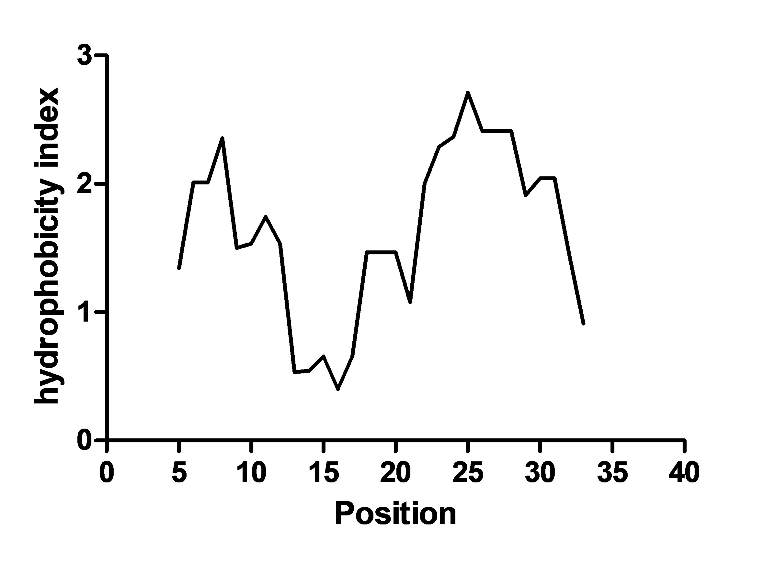
**

**Supplementary Figure 1. Hydrophobicity analysis of TM2 via the Kyte-Doolittle standard.** A hydrophobic distribution map of TM2 in EAAT2 was drawn according to the Kyte-Doolittle hydrophobicity standard (1982), taking nine consecutive amino-acid residues as a group of comprehensive hydrophobic indexes. A larger positive value denotes a stronger hydrophobicity, whereas a smaller negative value denotes a stronger hydrophilicity.


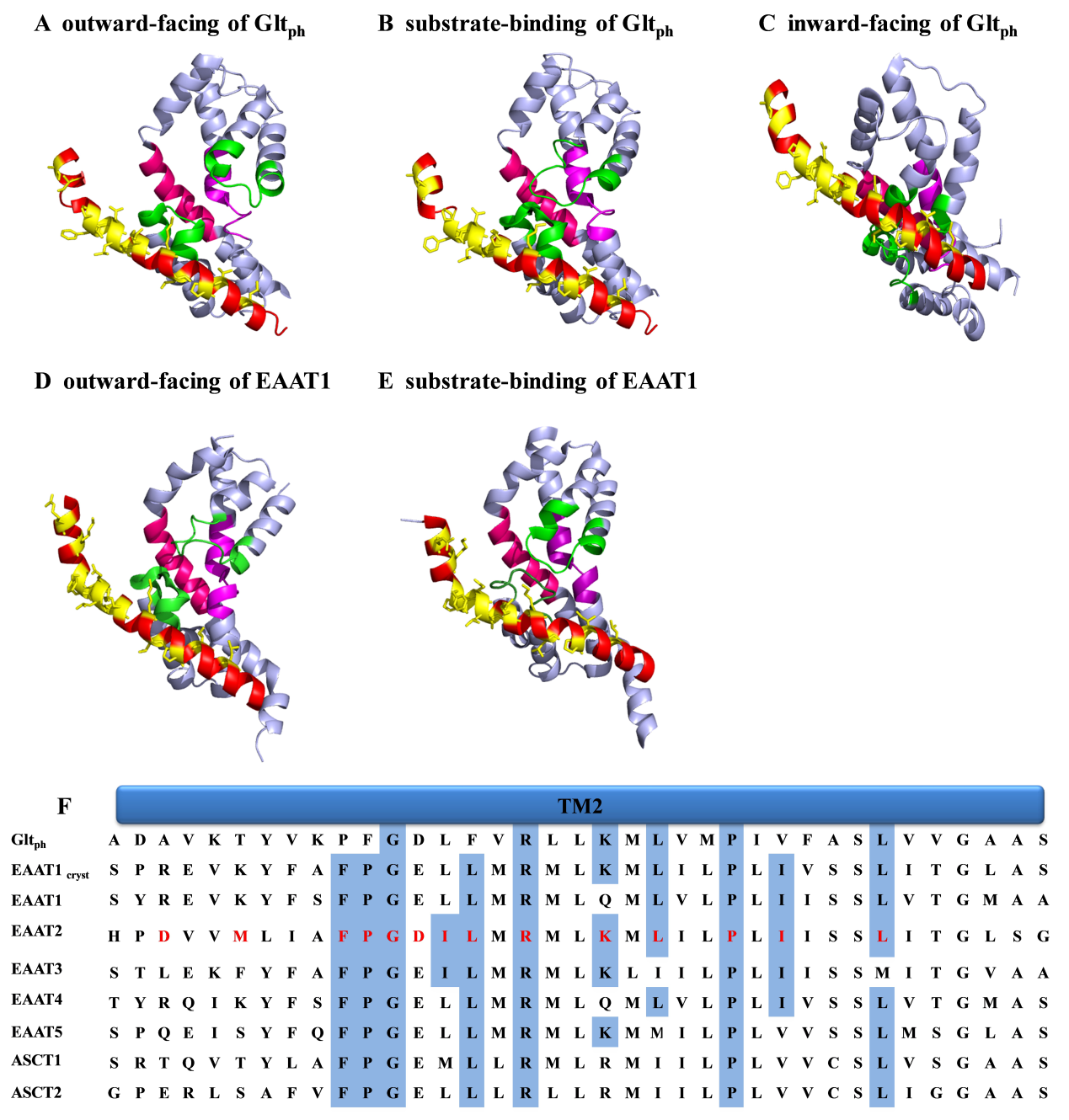


**Supplementary Figure 2. Structural relationship between TM2 and substrate binding sites and amino-acid sequence alignment of TM2.** The sequence homology of Glt_Ph_ and mammalian EAATs is approximately 37%*^28^*, indicating that this model is acceptable for studying the spatial relationship between substrate-binding pockets and TM2. The adjacent regions of substrate-binding sites are located in (A) Glt_Ph_ (PDB ID: 1XFH), (B) Glt_Ph_ (PDB ID: 2NWW), and (C) Glt_Ph_ (PDB ID: 3KBC). The thermostable EAAT1 (known as EAAT_cryst_) shares approximately 75% homology with wild-type EAAT1 and 90% homology at the transport core of the protein*^[29](#_ENREF_29)^*. (D) EAAT_cryst_(PDB ID: 5MJU) is shown. (E) EAAT_cryst_ (PDB ID: 5LLU) is shown. The protein regions in HP1, HP2, TM7, and TM8 are thought to be substrate-binding regions, which are highlighted in different colors. TM2 is shown in red, the 14 alanine-scanning mutation sites are shown in yellow, and HP1 & HP2 are shown in green. (F) Multiple sequence alignment of TM2s from Glt_Ph_, EAAT_cryst_, EAAT1–5, and ASCT1–2. The red-labeled amino acid sequences denote the fourteen critical residues of EAAT2 TM2 and are compared with those of other transporters in the same position.
